# Short- to mid-term outcomes after transcatheter aortic valve replacement in patients with ascending aorta dilation: a single-centre retrospective analysis

**DOI:** 10.1186/s12872-023-03063-8

**Published:** 2023-01-18

**Authors:** Jun Yu, Wei Wang

**Affiliations:** Department of Structural Heart Disease Centre, Fu Wai Hospital, National Center for Cardiovascular Diseases, Chinese Academy of Medical Sciences and Peking Union Medical College, A 167, Beilishi Road, Xicheng District, Beijing, 100037 China

**Keywords:** Transcatheter aortic valve replacement, Ascending aorta dilation, Self-expandable valve

## Abstract

**Objectives:**

Ascending aorta dilation (AAD) is frequently associated with aortic stenosis (AS). This study investigated the procedural and clinical outcomes of transcatheter aortic valve replacement (TAVR) in patients with AAD for tricuspid AS.

**Methods:**

This is a single-centre retrospective study that included patients with tricuspid AS and who underwent TAVR between January 1, 2018 and December 31, 2021. A total of 239 patients met the inclusion criteria. The ascending aortic diameter was measured on computed tomography (CT) scans before TAVR, and AAD was identified as a maximal ascending aortic diameter of ≥ 40 mm. The outcomes were in line with the Valve Academy Research Consortium (VARC)-3 criteria.

**Results:**

Self-expandable (SE) valves were used in 88.7% of the total cohort (89.0% in the AAD group and 88.6% in the non-AAD group). Seventy-three patients (30.5%) were diagnosed with concomitant AAD (mean age 73.7 ± 7.3 years, 57.5% male). The median ascending aortic diameter was 36.0 mm (interquartile range [IQR]: 34.0–37.0 mm) in the non-AAD group and 44.0 mm (IQR: 42.0–46.0 mm) in the AAD group (*p* < 0.001). The baseline characteristics were comparable across the groups. No significant difference was observed in cumulative all-cause mortality at 30 days (2.4% vs. 1.4%, *p* = 0.609), 1 year (9.2% vs. 5.0%, *p* = 0.191), or 3 years (13.1% vs. 9.5%, *p* = 0.201) between the non-AAD and AAD groups. The device success rate was not different between the non-AAD and AAD groups (74.7% vs. 82.2%, *p* = 0.205). The multivariable analysis identified prior percutaneous coronary intervention, prior stroke, and length of intensive care unit as independent predictors of 3-year all-cause mortality among the total cohort.

**Conclusion:**

AAD does not appear to be associated with the procedural and mid-term clinical outcomes in patients undergoing TAVR.

## Introduction

Transcatheter aortic valve replacement (TAVR) is now widely used in the management of high-risk and inoperable patients with tricuspid aortic stenosis (AS) [1, 2]. Aortic stenosis sometimes occurs concurrently with ascending aorta dilation (AAD) [3, 4] Patients with AAD often require surgical correction to prevent aortic dissection or rupture [5, 6]. The American College of Cardiology (ACC) Foundation guidelines recommended concomitant surgery when the ascending aortic diameter was > 45 mm [6], which cannot be applied in the TAVR procedure. However, as the indications for TAVR expand, more patients with AAD are being referred to have the procedure [7]. Almost 1% of patients with aortic stenosis present with thoracic aortic aneurysm in America [8]. The safety and feasibility of TAVR for patients with AAD deserve to be illustrated.

Self-expandable (SE) valves are characterized by a longer stent frame design, and the radial forces of the device are present at both the inflow and outflow levels, which may cause them to be challenging to use in patients with AAD [9]. A previous study concluded that a larger AAD was the most relevant predictor of device failure after SE valve implantation [9]. In addition, the Society of Cardiovascular Computed Tomography (SCCT) guidelines recommend that the proximal ascending aortic diameter measured by multidetector CT (MDCT) should not exceed 40–43 mm for SE valves [10]. However, SE-valves were used in most of the patients with AAD in this study, and the procedural and clinical outcomes of these patients deserve attention.

We initially thought that the dilation of the ascending aorta may not affect the prognosis of TAVR patients according to prior studies. The aim of the present study was to evaluate the procedural and clinical outcomes in patients with AAD compared to those without AAD undergoing TAVR.

## Materials and methods

### Patient population

Patients with tricuspid AS undergoing TAVR between January 1, 2018 and December 31, 2021 at our institution were included in the present study. The TAVR indications were as follows: (I) patients with intermediate- to high-risk aortic valve disease and a Society of Thoracic Surgeons risk (STS) score > 4; (II) patients who were aged ≥ 65 years; (III) patients with no contraindication to anticoagulation; (IV) patients were not at the acute stage of cerebrovascular events; and (V) patients without coronary heart disease requiring simultaneous revascularization. The following patients were excluded: (I) patients with any previous cardiovascular surgery; (II) patients with a bicuspid aortic valve; (III) patients with pure aortic regurgitation; and (IV) patients with poor MDCT imaging quality. This study was approved by the Medical Ethics Review Committee of Fuwai Hospital, and the requirement for informed consent was waived by the Medical Ethics Review Committee of Fuwai Hospital for the nature of a retrospective analysis.

### Transcatheter aortic valve replacement procedure

TAVR was performed in a hybrid operating room by the cardiology team, and the patients were placed under general anaesthesia or were given local anaesthesia and were monitored by anaesthesiologists. All patients underwent TAVR after discussions with the multidisciplinary cardiology team, and the access site and the type of prosthesis were determined thereafter. Transfemoral access was preferred in all patients who met the criteria unless the prosthesis size and the calcification and atheroma of the aorto-iliofemoral artery were considered. All TAVR procedures were performed according to the established standards via the transfemoral, trans-carotid, or trans-subclavian approach and by using contemporary devices, such as the implantation of SE valves (Venus-A [Venus Medtech, Hangzhou, China], VitaFlow [Microport, Shanghai, China], TaurusOne [Peijia Medical, Suzhou, China]) [11–13] or balloon expandable (BE) valves (Sapien 3 [Edwards Lifesciences, Irvine, California], Sapien XT [Edwards Lifesciences, Irvine, California]) [14, 15].

### Data collection

All patients underwent transthoracic echocardiography (TTE) and MDCT pre-TAVR. MDCT images were reconstructed with mid-systolic data using 3MENSIO Valves software (version 9.0, 3mensio Medical Imaging BV, Bilthoven, the Netherlands) [16] and were analysed by a dedicated core laboratory that included personnel who were blinded to the patient information and outcome data at our institution. The ascending aorta height was defined as the cross-sectional area 40 mm above the plane of the aortic annulus, as previously described [10]. The ascending aortic diameter was measured by the following method: (short axis aortic diameter + long axis aortic diameter)/2. Ascending aorta dilation was defined as a maximal ascending aortic diameter of ≥ 40 mm [4, 17]. The baseline characteristics and procedural and hospitalization data were retrospectively recorded manually from the institutional electronic medical record system and were entered into a dedicated database. All data were anonymized, systematically collected and assessed for quality.

### Follow-up and end points

All endpoints in this study were defined in accordance with the Valve Academic Research Consortium (VARC)-3 criteria [18]. The primary end point of the study was 3-year all-cause mortality. The secondary endpoints were 30-day and 1-year all-cause mortality. Other endpoints included technical success, device success, all stroke, myocardial infarction, VARC type ≥ 2 bleeding, new permanent pacemaker implantation, aortic reintervention or surgery, rehospitalization, acute kidney injury (stage 2–4), paravalvular leakage, cardiac tamponade, and aortic dissection. The patients were followed up until May 31, 2022, by outpatient visits and telephone interviews. No patient was lost to follow-up in this study. The median follow-up time was 588 days (interquartile range [IQR]: 384 to 1014 days).

### Statistical analysis

All continuous variables were tested for normality using the Shapiro‒Wilk test. Continuous variables were presented as the mean ± standard deviation (SD) and compared using Student’s t test or as the medians (25th–75th quartile) and were compared using the Mann‒Whitney U test. Categorical variables are presented as numbers and percentages and were compared using the Chi-square test or Fisher’s exact test. Periprocedural, early and late mortality were calculated using Kaplan‒Meier survival analysis, and the log-rank test was used for comparisons between the groups. To identify independent predictors of 3-year all-cause mortality among the total cohort, all variables with a *p* value < 0.10 in the univariate analysis were included in a stepwise multivariable Cox regression model. The proportional hazard assumption was confirmed by examination of log (−log [survival]) curves and by testing of partial (Schoenfeld) residuals, and no relevant violations were found. The estimated hazard ratio (HR) with 95% confidence interval (CI) was provided by Cox regression analysis. All tests were 2-sided, and *p* values < 0.05 were considered to be statistically significant. All statistical analyses were performed using SPSS software version 26.0 (IBM, NY, USA) and R software version 4.1.0 (available at http://www.r-project.org).

## Results

### Baseline characteristics

**Fig. 1:**
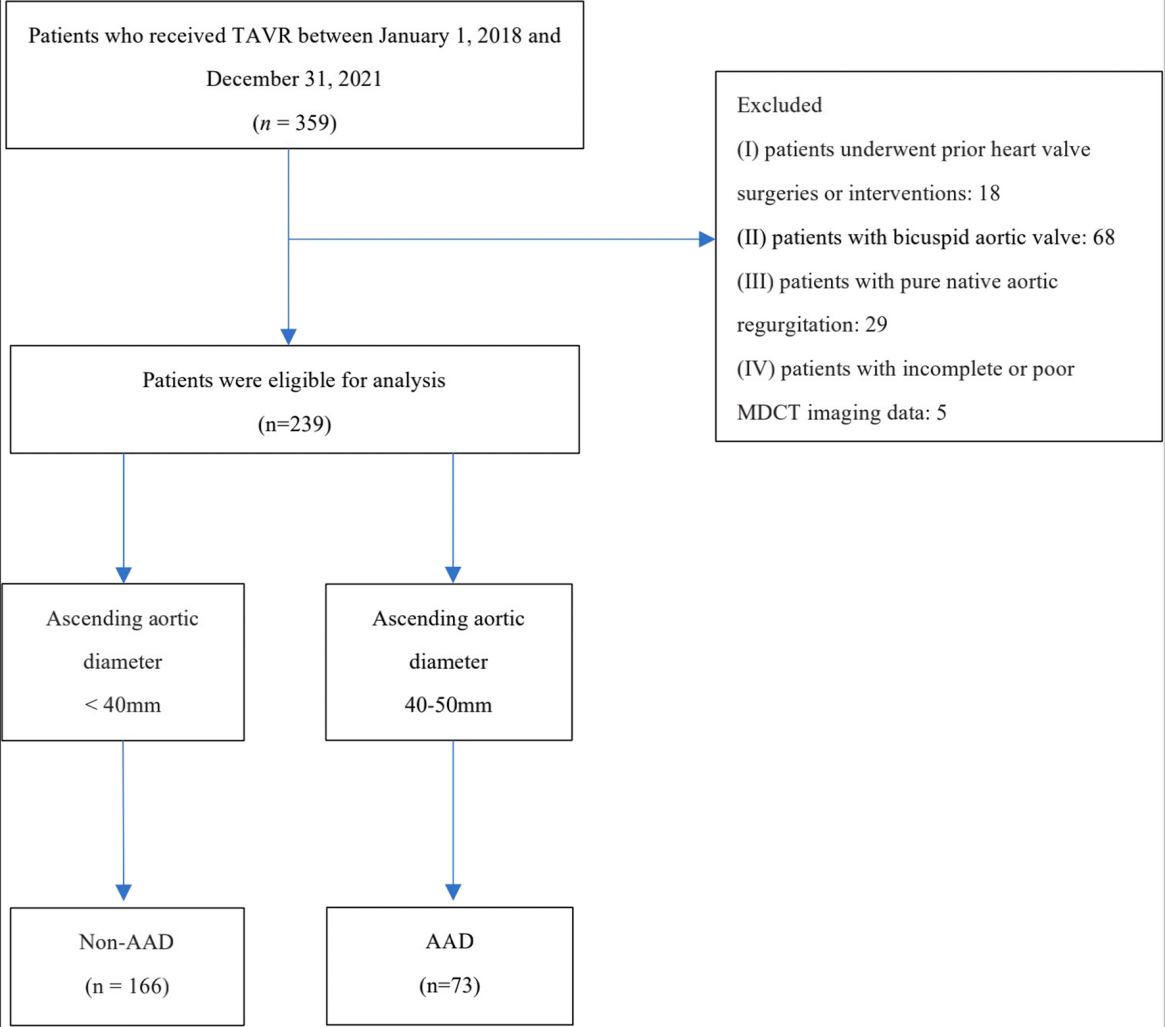
The flowchart of patient inclusion and exclusion. TAVR, transcatheter aortic valve replacement; MDCT, multidetector computed tomography; AAD, ascending aorta dilation

A total of 359 patients underwent TAVR during the study period at our institution (Fig. 1). A total of 120 patients were excluded according to the criteria (18 patients underwent previous heart valve surgeries or had interventions, 68 patients had a bicuspid aortic valve, 29 patients had pure native aortic regurgitation, and 5 patients had incomplete or poor MDCT imaging data). Therefore, a total of 239 patients with tricuspid AS were included in the present study. Seventy-three patients were diagnosed with AAD (mean age 73.7 ± 7.3, 57.5% male) and had an ascending aortic diameter of 40–50 mm. A total of 166 patients were diagnosed with non-AAD (mean age 73.1 ± 7.3, 51.8% male). The baseline characteristics were well balanced across the two groups (Table 1). Most patients were severely symptomatic (71.2% of the AAD group and 69.3% of the non-AAD group were NYHA functional class III or IV). The patients were at an intermediate risk as predicted by the STS score (median and interquartile range: 6.0 [5.0–7.0] % in the non-AAD group and 6.0 [5.0–7.0] % in the AAD group, *p* = 0.901). The haemodynamic severity and proportion of patients with aortic calcification of tricuspid AS were comparable between the two groups according to the echocardiographic and MDCT assessments. The ascending aortic diameters measured by MDCT were different (median and interquartile range: 36.0 [34.0–37.0] mm in the non-AAD group and 44.0 [42.0–46.0] in the AAD group, *p* < 0.001).

**Table 1:** Baseline characteristics

	Non-AAD (n = 166)	AAD (n = 73)	*p* value
Age, years	73.1 ± 7.3	73.7 ± 7.3	0.607
Male	86 (51.8)	42 (57.5)	0.102
BMI, kg/m^2^	25.2 ± 3.8	24.2 ± 3.1	0.058
NYHA functional class III or IV	115 (69.3)	52 (71.2)	0.761
Society of Thoracic Surgeons risk score, %	6.0 (5.0–7.0)	6.0 (5.0–7.0)	0.901
Prior history			
PCI	26 (15.7)	13 (17.8)	0.679
CABG	8 (4.8)	0 (0.0)	0.110
Stroke	24 (14.5)	6 (8.2)	0.180
Permanent pacemaker	1 (0.6)	2 (2.7)	0.222
Cancer	11 (6.6)	4 (5.5)	1.000
Comorbidities			
Hypertension	102 (61.4)	41 (56.2)	0.443
Diabetes mellitus	41 (24.7)	17 (23.3)	0.815
Hyperlipidaemia	68 (41.0)	31 (42.5)	0.828
Chronic obstructive pulmonary disease	8 (4.8)	0 (0.0)	0.110
Peripheral vascular disease	27 (16.3)	6 (8.2)	0.097
Heart failure	14 (8.4)	5 (6.8)	0.677
Chronic kidney disease	7 (4.2)	3 (4.1)	1.000
Liver disease	2 (1.2)	2 (2.7)	0.588
Heart failure	14 (8.4)	5 (6.8)	0.677
Coronary artery disease	77 (46.4)	24 (32.9)	0.051
Arrhythmia			
Atrial fibrillation	21 (12.7)	12 (16.4)	0.434
Other type of arrhythmia	12 (7.2)	10 (13.7)	0.111
CT characteristics			
Ascending aortic diameter (mm)	36.0 (34.0–37.0)	44.0 (42.0–46.0)	< 0.001
Aortic calcification			0.802
Grade I (none)	0 (0.0)	0 (0.0)	
Grade II (mild)	33 (19.9)	17 (23.3)	
Grade III (moderate)	107 (64.5)	44 (60.3)	
Grade IV (severe)	26 (15.7)	12 (16.4)	
Echocardiographic assessment			
Left ventricular ejection fraction, %	60.0 (50.0–65.0)	60.0 (46.5–65.0)	0.719
Maximum velocity, m/s	4.6 (4.1–5.0)	4.8 (4.3–5.2)	0.065
Mean aortic valve gradient, mmHg	50.0 (41.0–63.3)	54.0 (42.0–67.0)	0.318
Aortic regurgitation ≥ moderate	44 (26.5)	22 (30.1)	0.563
Mitral regurgitation ≥ moderate	25 (15.1)	5 (6.8)	0.078
Tricuspid regurgitation ≥ moderate	17 (10.2)	3 (4.1)	0.115
Pulmonary hypertension	39 (23.5)	10 (13.7)	0.084
Hospitalization length			
Intensive care unit (days)	1.0 (1.0–3.0)	1.0 (1.0–2.0)	0.175
Total length (days)	12.0 (9.0–18.0)	12.0 (9.5–15.0)	0.625

Values are mean ± SD, n (%), or median (interquartile range). *AAD* ascending aorta dilation; *BMI* body mass index; *PCI* percutaneous coronary intervention; *CABG* coronary artery bypass graft; *NYHA* New York Heart Association; *CT* computed tomography

### Procedural data and results

The procedural data and results are summarized in Table 2, and no significant differences were observed between the non-AAD and AAD groups. Emergency surgery was performed in 9 patients (5.4%) in the non-AAD group and in 2 patients (2.7%) in the AAD group. A total of 137 patients (82.5%) in the non-AAD group and 60 patients (82.2%) in the AAD group were under general anaesthesia during the procedure. The most frequent access site was via the transfemoral approach (96.4% of the patients in the non-AAD group and 94.5% of the patients in the AAD group). An SE-valve was used in the majority of the patients undergoing TAVR, and it was used in 147 patients (88.6%) in the non-AAD group and 65 patients (89.0%) in the AAD group. Balloon postdilation was more common in the AAD group (23.3% vs. 12.0, *p* = 0.027). Postprocedural aortic regurgitation grade ≥ moderate was present in 2 patients (1.2%) in the non-AAD group and 1 patient (1.4%) in the AAD group. Six patients (3.6%) in the non-AAD group and 1 patient (1.4%) in the AAD group underwent conversion to open surgery. The proportion of patients who underwent implantation of a second valve was not significantly different between the non-AAD group (30 patients, 18.1%) and the AAD group (10 patients, 13.7%, *p* = 0.404). The left ventricular ejection fraction at discharge was not different between the non-AAD group and AAD group (*p* = 0.435).

**Table 2:** Procedural data and results

	Non-AAD (n = 166)	AAD (n = 73)	*p* value
Emergency surgery	9 (5.4)	2 (2.7)	0.511
General anaesthesia	137 (82.5)	60 (82.2)	0.950
Access			
Transfemoral	160 (96.4)	69 (94.5)	0.755
Non-transfemoral (Carotid, Subclavian)	6 (3.6)	4 (5.5)	
Valve type			
Self-expandable valve (Venus-A, VitaFlow, TaurusOne)	147 (88.6)	65 (89.0)	0.767
Balloon-expandable valve (Sapien3, Sapien XT)	19 (11.4)	8 (11.0)	
Balloon pre-dilation	149 (89.8)	63 (86.3)	0.437
Balloon post-dilation	20 (12.0)	17 (23.3)	0.027
Post-procedure AR grade			
≥ mild	64 (38.6)	29 (39.7)	0.864
≥ moderate	2 (1.2)	1 (1.4)	1.000
Conversion to open surgery	6 (3.6)	1 (1.4)	0.679
Second valve implantation	30 (18.1)	10 (13.7)	0.404
Device migration to ventricle	20 (12.0)	4 (5.5)	
Paravalvular leakage	6 (3.6)	4 (5.5)	
Device expand inadequately	4 (2.4)	1 (1.4)	
Left ventricular ejection fraction at discharge, %	60.0 (55.0–65.0)	60.0 (50.0–65.0)	0.435

Values are mean ± SD, n (%), or median (interquartile range). *AAD* ascending aorta dilation

### Clinical outcomes

**Fig. 2:**
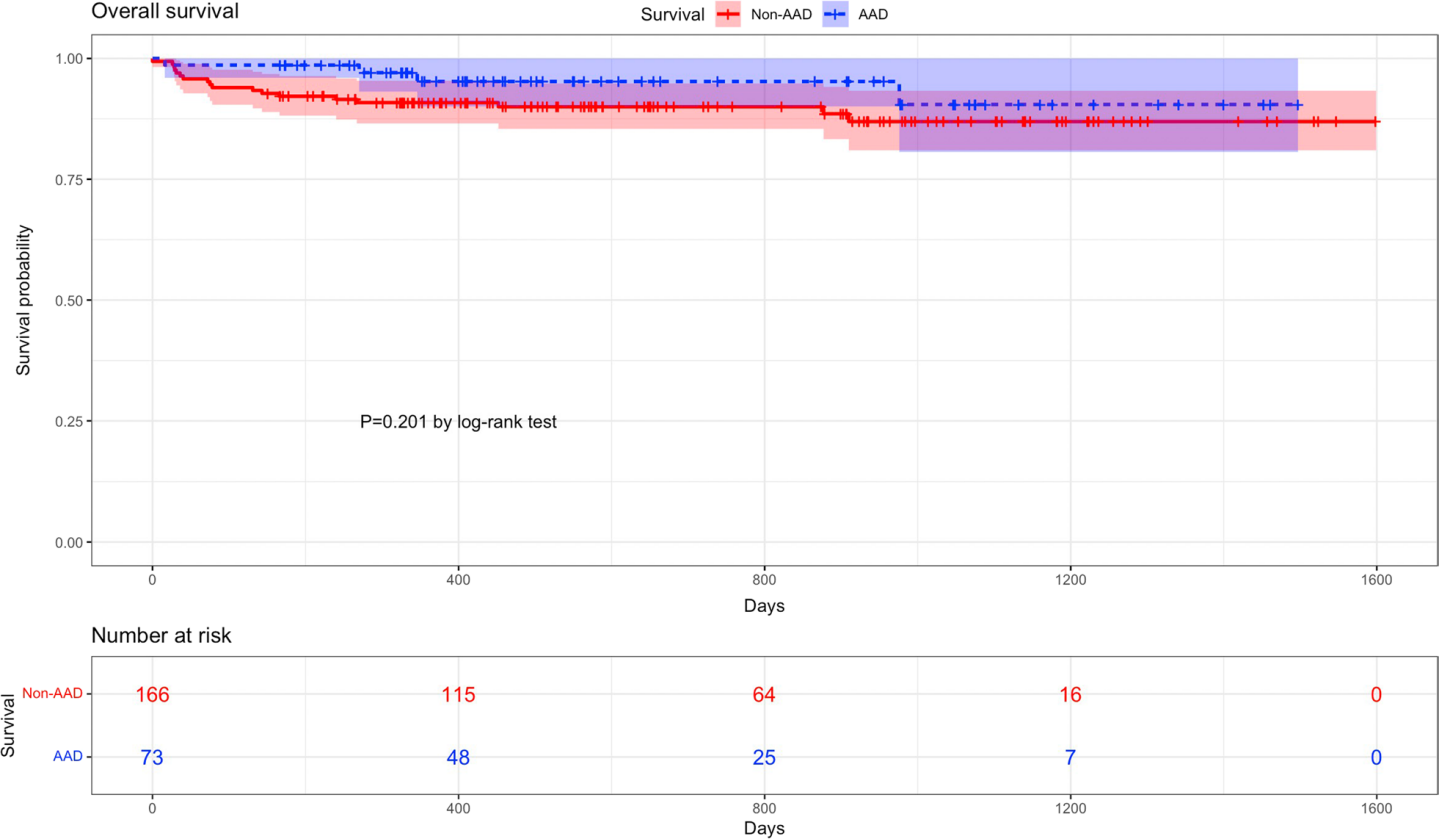
Kaplan‒Meier survival curves of all-cause mortality between the non-AAD and AAD groups. AAD, ascending aorta dilation

The technical success was 80.1% in the non-AAD group and 84.9% in the AAD group, but no significant difference was observed (*p* = 0.377). The device success was 74.7% in the non-AAD group and 82.2% in the AAD group (*p* = 0.205). Cumulative all-cause mortality at 30 days, 1 year, and 3 years was 2.4%, 9.2%, and 13.1% in the non-AAD group and 1.4%, 5.0%, and 9.5% in the AAD group, respectively. There were no significant differences in periprocedural mortality, early mortality, and late mortality between the non-AAD and AAD groups. The causes of mortality are shown in Table 3. Kaplan‒Meier survival curves of all-cause mortality between the two cohorts are shown in Fig. 2. Other clinical endpoints, such as all stroke, myocardial infarction, VARC type ≥ 2 bleeding, new permanent pacemaker implantation, aortic reintervention or surgery, rehospitalization, significant paravalvular leakage, and acute kidney injury (stage 2–4), were also comparable between the groups. Cardiac tamponade and aortic dissection were not observed in either group (Table 3).

**Table 3:** Clinical outcomes

	Non-AAD (n = 166)	AAD (n = 73)	*p* value
Technical success (at exit from procedure room)	133 (80.1)	62 (84.9)	0.377
Device success (at 30 days)	124 (74.7)	60 (82.2)	0.205
Periprocedural mortality (at 30 days)			
All-cause mortality	4 (2.4)	1 (1.4)	0.609
Cardiovascular mortality	3 (1.8)	0 (0.0)	0.251
Valve-related mortality	1 (0.6)	0 (0.0)	0.509
Early mortality (at 1 year)			
All-cause mortality	15 (9.2)	3 (5.0)	0.191
Cardiovascular mortality	12 (7.7)	1 (2.0)	0.072
Valve-related mortality	3 (1.9)	0 (0.0)	0.245
late mortality (at 3 years)			
All-cause mortality	18 (13.1)	4 (9.5)	0.201
Cardiovascular mortality	13 (10.0)	1 (1.9)	0.056
Valve-related mortality	3 (1.9)	0 (0.0)	0.245
All stroke	6 (3.6)	5 (6.8)	0.318
Myocardial infarction	5 (3.0)	1 (1.4)	0.670
VARC type ≥ 2 bleeding	9 (5.4)	3 (4.1)	0.915
New permanent pacemaker implantation	12 (7.2)	6 (8.2)	0.789
Aortic reintervention or surgery (structural valve deterioration)	1 (0.6)	2 (2.7)	0.222
Re-hospitalization	50 (30.1)	24 (32.9)	0.671
Cardiovascular hospitalization	37 (22.3)	15 (20.5)	
Non-cardiovascular hospitalization	13 (7.8)	9 (12.3)	
Acute kidney injury (stage 2–4)	1 (1.8)	1 (4.3)	0.500
Paravalvular leakage	1 (0.6)	2 (2.7)	0.222
Cardiac tamponade	0 (0.0)	0 (0.0)	–
Aortic dissection	0 (0.0)	0 (0.0)	–

Values are n (%). *AAD* ascending aorta dilation; *VARC* Valve Academy Research Consortium

In the multivariable analysis (Table 4), the factors that had been identified as independent predictors of 3-year all-cause mortality among the total cohort were prior percutaneous coronary intervention (HR: 2.92; 95% CI: 1.15 to 7.42; *p* = 0.024), prior stroke (HR: 3.42; 95% CI: 1.37 to 8.54; *p* = 0.009), and length of intensive care unit (HR per 1 day increase: 1.07; 95% CI: 1.04 to 1.10; *p* < 0.001).

**Table 4:** Predictive factors of all-cause mortality in the total cohort

	Univariate model		Multivariate model	
	HR (95% CI)	*p* value	HR (95% CI)	*p* value
Prior PCI	2.35 (0.96–5.75)	0.063	2.92 (1.15–7.42)	0.024
Prior stroke	3.28 (1.33–8.08)	0.010	3.42 (1.37–8.54)	0.009
Hyperlipidaemia	2.93 (1.19–7.21)	0.019		
Peripheral vascular disease	2.24 (0.87–5.74)	0.095		
Heart failure	3.04 (1.02–9.02)	0.045		
Coronary artery disease	4.68 (1.73–12.70)	0.002		
Emergency surgery	3.65 (1.08–12.35)	0.038		
Conversion to open surgery	6.68 (1.96–22.72)	0.002		
Total length, per increase of 1 day	1.04 (1.01–1.06)	0.004		
Intensive care unit, per increase of 1 day	1.06 (1.03–1.09)	< 0.001	1.07 (1.04–1.10)	< 0.001

*PCI* percutaneous coronary intervention, *HR* hazard ratio, *CI* confidence interval

## Discussion

The key findings of our study were as follows: patients with AAD accounted for almost one-third of the overall population; the prevalence of patients with SE-valve was 88.6% in the non-AAD group and 89.0% in the AAD group, which are significantly higher proportions than those that have been previously reported for patients with TAVR [19]; none of the procedural and clinical outcomes were associated with AAD.

AAD is a common disorder in patients with AS, occurring in 1/5 to 1/4 of these patients [20–22]. However, the proportion in the present study was higher, with nearly one-third of the patients with AAD undergoing TAVR. To date, open surgery remains the standard treatment for patients with AAD. It is widely accepted that endovascular repair can treat descending aortic pathologies; however, its use for aortic arch and ascending aortic pathologies remains undefined and limited [23]. The TAVR procedure concomitant with endovascular intervention for AAD is unknown. With the expanding indications for TAVR, an increasing number of patients with tricuspid AS and concomitant AAD may receive TAVR treatment, especially those with an intermediate or a high surgical risk [7]. TAVR is a more minimally invasive treatment, but whether patients with AAD can benefit from it and whether AAD should become a new powerful risk stratification criterion for TAVR need to be considered.

According to previous studies [24, 25], patients with tricuspid AS with a concomitant ascending aorta diameter of 40–50 mm are at low risk of adverse aortic events after isolated aortic valve replacement. A conservative therapy strategy for AAD at the time of aortic valve surgery was recommended. For patients undergoing TAVR, similar studies showed that AAD did not increase the intraprocedural risk of adverse aortic events, nor did it affect mid-term survival [7, 19]. However, these studies did not compare the causes of death and other clinical outcomes in detail, and selection bias might exist. In our study, the baseline characteristics of the two groups were well balanced and comparable, and no differences were observed in technical success, device success, periprocedural, early and late mortality. All these results suggested that dilation of the ascending aorta may not affect the prognosis of TAVR patients. However, another large observational study showed that AAD was an independent risk predictor for 2-year all-cause mortality [26], which is a result that was obviously different from other studies. This may be due to the large sample size of the study. It needs to be noted that the diameter of the ascending aorta in these studies was mainly between 40 and 50 mm. There is a lack of relevant research evidence for patients with ascending aortic diameter ≥ 50 mm. Therefore, caution should be exercised in extrapolating these conclusions, especially when the current findings still remain controversial.

In theory, the SE valve needs to be closely attached to the valvular-aortic complex at both the inflow and outflow levels to obtain a sufficient radial force to ensure the stability of the valve position and to avoid paravalvular leak and valve malposition [9]. Therefore, when using SE valves, it is difficult for the outflow stent frame to be closely attached to the dilated ascending aorta for patients with AAD, and because of this, the stability of the prosthesis will be greatly reduced after deployment, thereby increasing the risk of paravalvular leak and the need for a second valve, which may affect long-term prognosis. The CHOICE randomized clinical trial suggested that there was a significantly lower device success of patients with SE-valves than with BE-valves [27]. An observational study also showed that increased diameter of the ascending aorta was an independent predictor of unsuccessful device implantation of TAVR following the use of the SE-valve [9]. However, in this study, an SE-valve was used in nearly 90% of the patients in both groups, but there were no significant differences in the procedural and clinical outcomes between the AAD and non-AAD groups. Furthermore, valve type was not an independent predictor of 3-year all-cause mortality among the total cohort. Based on the results of our study, the SE-valve seems to be safe and feasible in patients with AAD. However, there are many factors such as the proportion of oversize, aortic root angulation and depth of the implanted valve that need to be corroborated by more detailed studies in the future.

## Study limitations

First, the study had the inherent limitations of a single-centre retrospective study, which could introduce potential selection bias and confounders. Second, the results must be interpreted with caution since the diameter of the ascending aorta was ≤ 50 mm and because patients with bicuspid aortic stenosis with AAD were excluded. However, patients with BAV are more prone to AAD, which could impact the balance of the two groups, and potential bias could exist. Third, we did not perform a propensity-score matching analysis due to the relatively small sample size, and the baseline characteristics were comparable between groups. Fourth, a potential limitation of the study is the significant difference between the patient group size of the non-AAD and AAD groups, which could potentially affect the mortality outcome statistics. Finally, device and prosthesis selection were not randomized but was at the operator’s discretion, and patient selection as well as the operator’s experience may have affected the clinical outcomes.

## Conclusion

AAD does not appear to be associated with the procedural and mid-term clinical outcomes in patients undergoing TAVR.

## Data Availability

The data that support the findings of this study are available from the corresponding author upon reasonable request.
